# Supplementary material for: Discrimination of Copper Molten Marks through a Fire Reproduction Experiment Using Microstructure Features
Source: Materials (Basel). 2022 Nov 18;15(22):8206. doi: 10.3390/ma15228206 (PMC9699099; doi:10.3390/ma15228206)
Supplement: Supplementary file 1 [file materials-15-08206-s001.zip › materials-2006587-supplementary.pdf]

materials

Supplementary Material

Discrimination of Copper Molten Marks through a Fire Reproduction Experiment Using Microstructure Features

Jinyoung Park [1], Joo-Hee Kang [2,*], Jiwon Park [2,*], Young Ho Ko [3] and Sun Bae Bang [1]

[1] Korea Electrical Safety Corporation Research Institute, 111, Anjeon-ro, Iseo-myeon, Wanju-gun 55365, Jeollabuk-do, Republic of Korea
[2] Korea Institute of Materials Science, 797, Changwon-daero, Seongsan-gu, Changwon-si 51508, Gyeongsangnam-do, Republic of Korea
[3] Jeonbuk National University, 567, Baekje-daero, Deokjin-gu, Jeonju-si 54896, Jeollabuk-do, Republic of Korea
* Correspondence: joohee@kims.re.kr (J.-H.K.); jiwonp@kims.re.kr (J.P.); Tel.: +82-55-280-3398 (J.-H.K.); +82-55-280-3372 (J.P.)

Supplementary information

Citation: Park, J.; Kang, J.-H.; Park, J.; Ko, Y.H.; Bang, S.B. Discrimination of Copper Molten Marks through a Fire Reproduction Experiment Using Microstructure Features. *Materials* **2022**, *15*, 8206. https://doi.org/10.3390/ma15228206

Academic Editor: Bolv Xiao

Received: 18 October 2022
Accepted: 16 November 2022
Published: 18 November 2022

Publisher's Note: MDPI stays neutral with regard to jurisdictional claims in published maps and institutional affiliations.

Copyright: © 2022 by the authors. Licensee MDPI, Basel, Switzerland. This article is an open access article distributed under the terms and conditions of the Creative Commons Attribution (CC BY) license (https://creativecommons.org/licenses/by/4.0/).

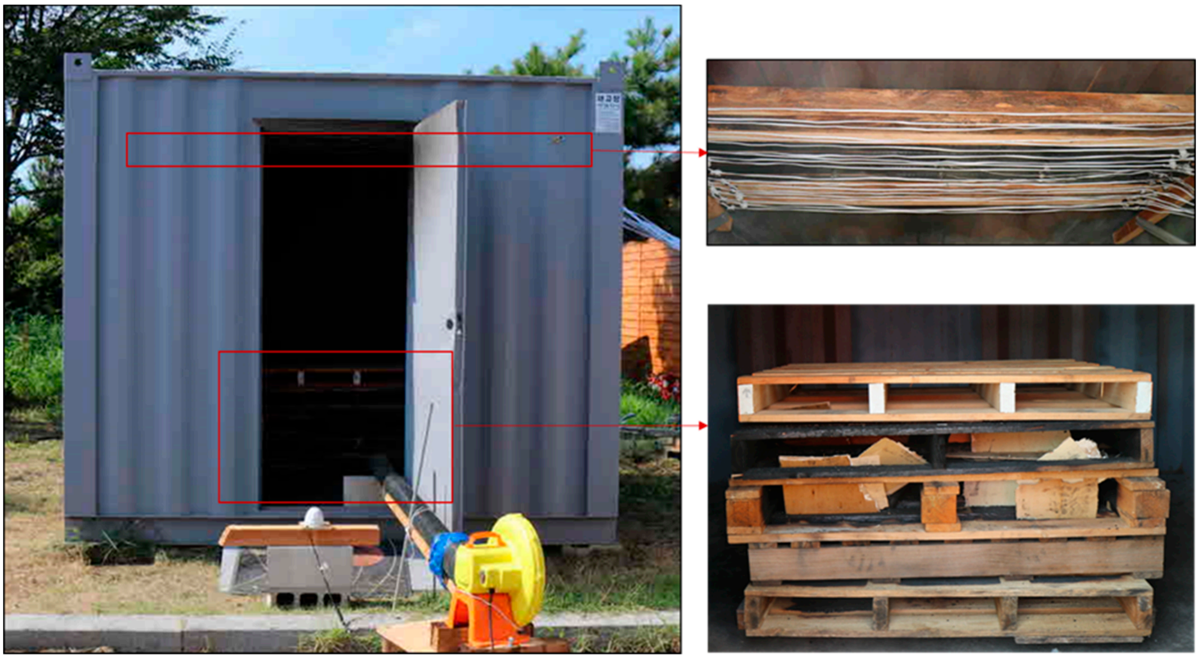

Figure S1. Test room configuration for the actual fire reproduction experiment.

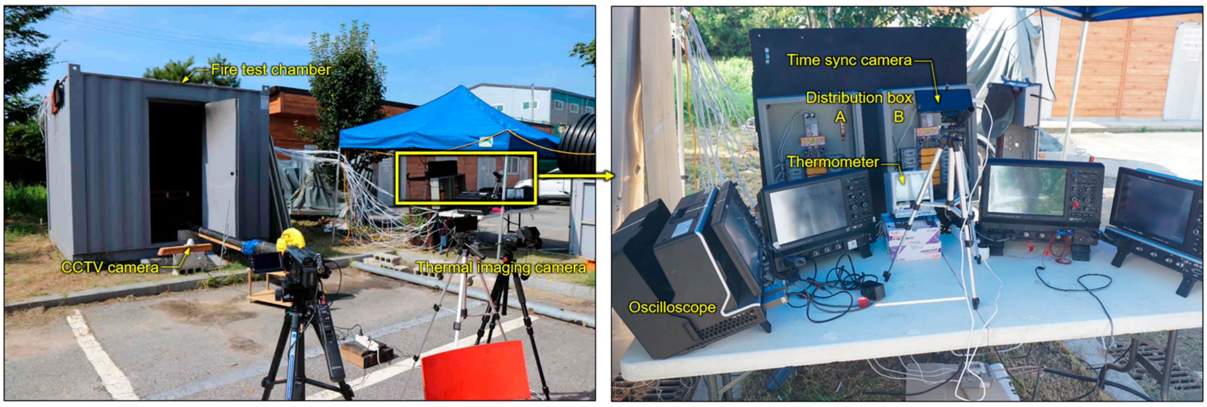

Figure S2. Configuration of the temperature, voltage, and short-circuit current measuring devices.

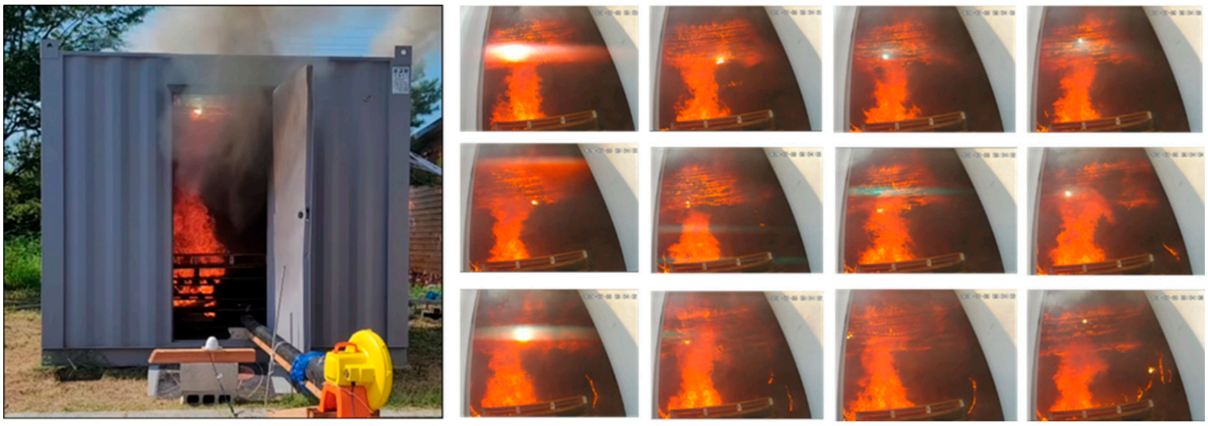

Figure S3. Identification of location of the short circuit. Short circuit occurs where the flame is in direct contact with the wire. The right images were captured by a CCTV camera in the yellow region in Fig. 3.

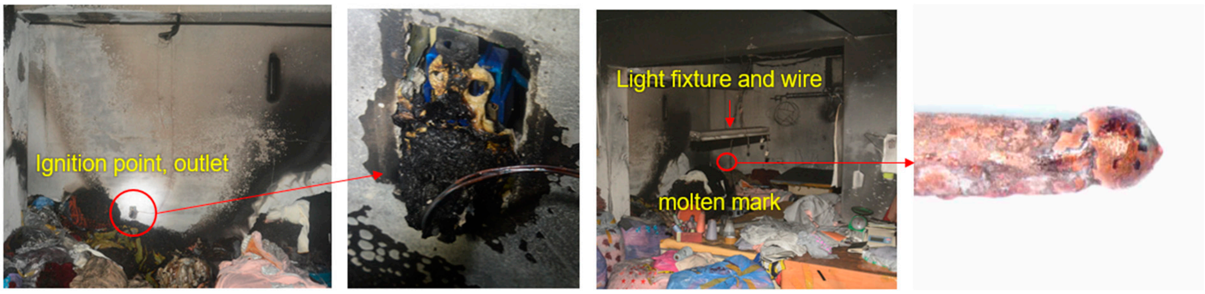

Figure S4. First case: actual fire in a textile workshop (Sample A).

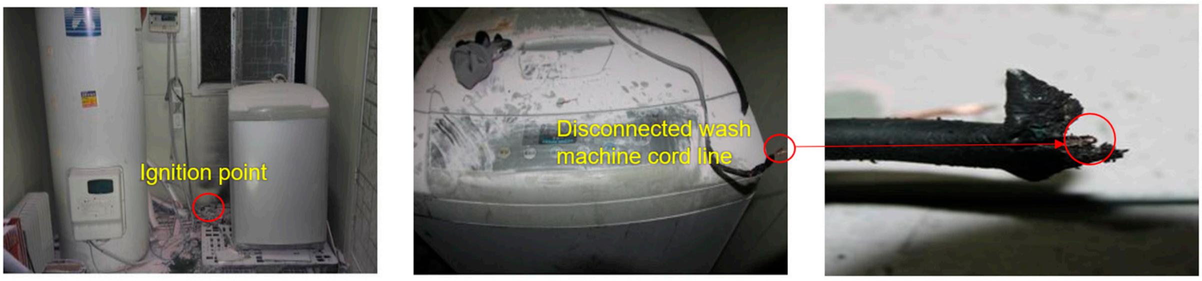

Figure S5. Second case: short circuit occurred in the electrical cord of the washing machine (Sample B).

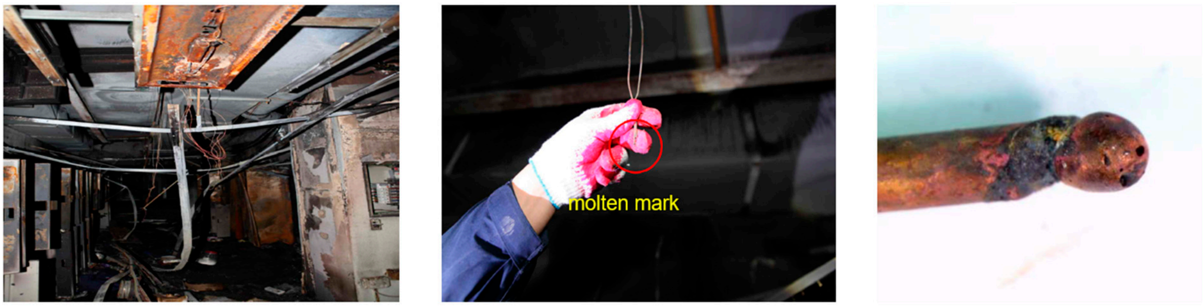

Figure S6. Third case: a short circuit occurred in a fluorescent lighting fixture (Sample C).

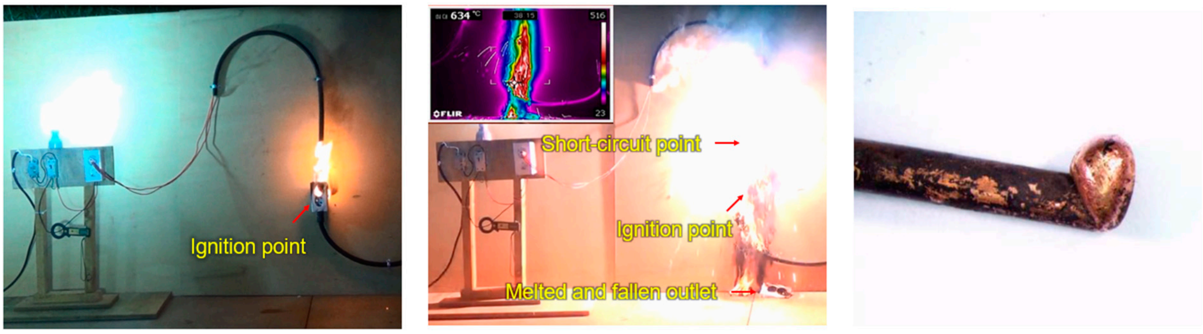

Figure S7. Fourth case: a short circuit occurred in the electric wire (Sample D).
